# Use of metallic and polymeric ureteral stents in malignant ureteral obstruction

**DOI:** 10.1002/bco2.51

**Published:** 2020-10-15

**Authors:** Mari Ohtaka, Takashi Kawahara, Yutaro Hayashi, Ryosuke Kobayashi, Sohgo Tsutsumi, Kimito Ousaka, Akitoshi Takizawa, Takeshi Kishida, Masahiro Yao, Hiroji Uemura

**Affiliations:** ^1^ Departments of Urology and Renal Transplantation Yokohama City University Medical Center Yokohama Japan; ^2^ Department of Urology Yokohama City University Graduate School of Medicine Yokohama Japan; ^3^ Department of Urology International Goodwill Hospital Yokohama Japan; ^4^ Department of Urology Kanagawa Cancer Center Yokohama Japan

**Keywords:** malignant ureteral obstruction, ureteral stenting

## Abstract

**Background:**

Malignant ureteral obstruction (MUO) is often caused by advanced intra‐abdominal cancers. Effective management must be attempted, but the treatment policy is unclear. Metallic ureteral stents are one of the latest options in managing MUO. Metallic ureteral stents are superior to traditional polyurethane stents. The present study retrospectively reviewed our four institutions’ experiences with treating MUO using metallic ureteral stent.

**Methods:**

A total of 45 patients who required metallic ureteral stent placement for MUO at Yokohama City University Medical Center (Yokohama, JAPAN) between January 2014 and May 2016 were analyzed. We defined stent failure as having to change the ureteral stent before the scheduled ureteral stent exchange time or having to perform percutaneous nephrostomy (PCN). Complications were defined as an unscheduled hospital visit or hospitalization caused by incompatibility, infection, and pain of the metallic ureteral stent, etc., unrelated to the primary disease. We compared stent failure and the overall survival (OS) between metallic and polymeric ureteral stents. To evaluate the workload of the medical staff, we used the NASA Task Load Index (NASA‐TLX) in a total of 11 urologists.

**Results:**

During the observation period, 8 (17.8%) patients in the metallic ureteral stent group and 10 (27.8%) in the control group developed stent failure. Complications were noted in 14 (31.1%) patients in the metallic ureteral stent group and 15 (41.7%) patients in the control group. A Kaplan–Meier analysis and log‐rank test showed no significant differences between two groups in the overall survival (*P* = 0.673). One or more complications developed in 19 (32.2%) patients in the metallic ureteral stent group and 18 (38.3%) patients in the control group (*P* = 0.409). Renal dysfunction after the replacement of the ureteral stent developed in 9 (15.3%) patients in the metallic ureteral stent group and 14 (29.8%) patients in the control group. No patients developed a urinary tract infection (UTI) that required hospitalization in the metallic ureteral stent group, whereas 3 (6.4%) patients in the control group had a UTI that was treated with hospitalization. The average workload score in the six subscales was analyzed, and the scores for mental demand and performance were higher in the metallic ureteral stent group, although there was no significant difference between the metallic and polymeric ureteral stent groups.

**Conclusions:**

Metallic ureteral stents showed favorable ureteral stent patency and reduced the workload for urologists.

## 1 BACKGROUND

Malignant ureteral obstruction (MUO) is often caused by advanced intra‐abdominal cancers. Effective management must be attempted, but the treatment policy is unclear.^1^, ^2^, ^3^, ^4^, ^5^, ^6^, ^7^ The current management options are retrograde ureteral stent (RUS) placement or percutaneous nephrostomy under local anesthesia. RUS is usually considered as the first treatment choice because of its low invasiveness. However, the stent failure rate is high, with a mean failure rate of 12.2%–34.6%,^8^, ^9^, ^10^, ^11^, ^12^, ^13^ and stents must be exchanged every 3 months.^14^

Resonance metallic ureteral stents (Cook Medical, Bloomington, IN, USA) are one of the latest options for managing MUO. Metallic ureteral stents are superior to traditional polyurethane stents (hereafter called “polymeric ureteral stents”) with respect to endurance against external force and frequency of exchange procedures.^15^ Indeed, metallic stents need be replaced only once a year, which can improve patients’ quality of life and relieve the workload of medical staff.

Some authors have reported the outcomes of metallic ureteral stents and the risk factors of stent failure, including prior radiotherapy, genitourinary cancer (GU) cancer, and urinary tract infections (UTIs).^16^, ^17^ Po‐Ming et al. said that metallic stents provide a longer functional duration than polymeric ureteral stents and should be offered as an option for internal drainage.^18^ However, few studies have compared the complications and cost. And no study examined the stress of metallic ureteral stenting procedure for urologist.

The present study retrospectively reviewed our four institutions’ experiences with treating MUO using metallic ureteral stents especially in tolerance of metallic stent and work load for stenting procedure.

## 2 MATERIALS AND METHODS

A total of 45 patients (59 ureters) who required metallic ureteral stent placement for MUO at Yokohama City University Medical Center (Yokohama, Japan) between January 2014 and March 2018 were retrospectively collected. As a control group, 36 patients (47 ureters) who received polymeric ureteral stents for MUO at Yokohama City University Medical Center between January 2014 and May 2016 were also analyzed (Table 1).

**TABLE 1:** Patients' background

	Polyurethane stent	Metallic stent
	n (%) or (median, range)	
Age (median, range)	70 (40–89)	68 (45–89)
Gender		
Male	12 (33.3)	19 (42.2)
Female	24 (66.7)	26 (57.8)
Observational days (median, range)	184 (5–2022)	183 (9–1145)
Primary disease		
Gastrointestinal cancer	17 (47.2)	22 (48.9)
Gynecological cancer	14 (38.8)	13 (28.9)
Lung cancer	1 (2.8)	2 (4.4)
Urological cancer	2 (5.6)	7 (15.6)
Unknown site cancer	2 (5.6)	1 (2.2)

Primary indwelling metallic ureteral stent placement was indicated for a variety of reasons, including pain control of hydronephrosis and improvement of the renal function, chemotherapy, as well as for polymeric stent failure. After introducing metallic ureteral stent from 2014, metallic ureteral stent was inserted first for the case who were MUO or ureteral stenosis come from benign tumor. The case with ureteral stone or the case who have ureteral infection was performed polyurethane ureteral stenting. At our institutions, all ureters underwent RUS with a rigid cystoscope under fluoroscopic guidance; 41 ureters (69.5%) were examined under local anesthesia, and 18 ureters (30.5%) were examined under general anesthesia. We usually exchanged the stent every 12 months. In the control group, all ureters underwent RUS with a rigid cystoscope under fluoroscopic guidance with local anesthesia; a 6‐Fr ureteric stent (Polaris^TM^ Ultra, Boston Scientific, Natick, MA, USA) with a 0.035‐mm guide wire (SENSOR^TM^, Boston Scientific) was used, and the stent was changed every 3 months. Renal function was assessed Cockcroft–Gault formula.

We defined stent failure as having to change the ureteral stent before the scheduled ureteral stent exchange time or having to perform percutaneous nephrostomy (PCN). Unscheduled ureteral stent exchange were performed the cases who suspected pyelonephritis by CT. Complications were defined as an unscheduled hospital visit or hospitalization caused by incompatibility, infection or pain of the metallic ureteral stent, etc., unrelated to worsening of the primary disease. We compared stent failure and the overall survival (OS) between metallic and polymeric ureteral stents. We also analyzed the complications experienced with metallic and polymeric ureteral stent. Patients’ age, gender, type of cancer, pre‐stenting, history of chemotherapy and radiotherapy, and local infiltration were assessed as predictive factors for complications.

In terms of the cost, we assumed that all ureters underwent RUS with local anesthesia and that the median survival time was 2 years. We compared the cost between metallic and polymeric ureteral stents at 2 and 2.5 years after insertion of an indwelling ureteral stent. To evaluate the workload of the medical staff, we used the NASA Task Load Index (NASA‐TLX).^19^, ^20^ The NASA‐TLX is widely regarded as the gold standard for measuring subjective workload. It provides an overall workload score based on a weighted average of ratings on six subscales: Mental Demands, Physical Demands, Temporal Demands, Performance, Effort, and Frustration. We also compared the workload score between metallic and polymeric ureteral stent in a total of 11 urologists.

### 2.1 Statistical analyses

Univariate and multivariate logistic regression analyses were performed to determine the predictors of complications. Odds ratios (ORs) were computed along with 95% confidence intervals (CIs). The survival duration was defined as the time between the date of RUS placement and death. A log‐rank test was performed for comparisons between metallic ureteral stent and polymeric ureteral stent groups. *P*‐values of < .05 were considered to indicate statistical significance. All statistical analyses were performed using the EZR software program (Saitama, Japan) and GraphPad Prism software program (GraphPad Software, La Jolla, CA, USA).

## 3 RESULTS

### 3.1 Patients’ characteristics and outcomes

The 45 patients included 19 males and 26 females. The median age (range) was 68.0 (45–89) years, and the median observation period (range) was 183 (9–1145) days. The causative diseases are summarized in Table 1 and included gastrointestinal cancer, gynecological cancer, lung cancer, and urological cancer. In the control group, the 34 patients included 12 males and 24 females. The median age (range) was 70.0 (40–89) years, and the median observation period (range) was 184 (5–2022) days. During the observation period, 8 (17.8%) patients in the metallic ureteral stent group and 10 (27.8%) patients in the control group had stent failure. Complications were experienced by 14 (31.1%) patients in the metallic ureteral stent group and 15 (41.7%) patients in the control group. In terms of complication, younger age (<60) group showed significantly higher than elderly (≥60) group (Table 5).

**FIGURE 1:**
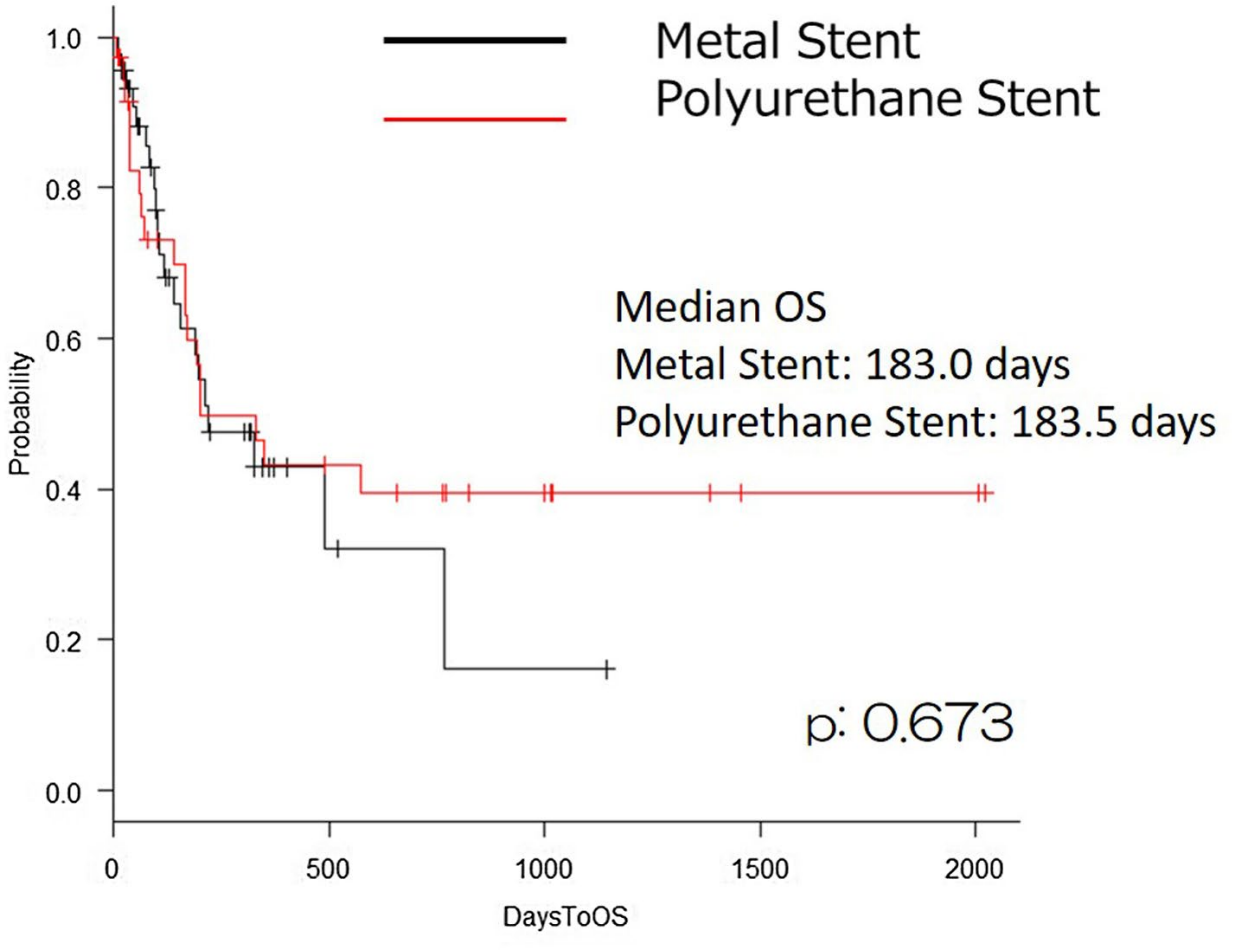
Overall survival in patients with metal stent or polyurethane stent

**FIGURE 2:**
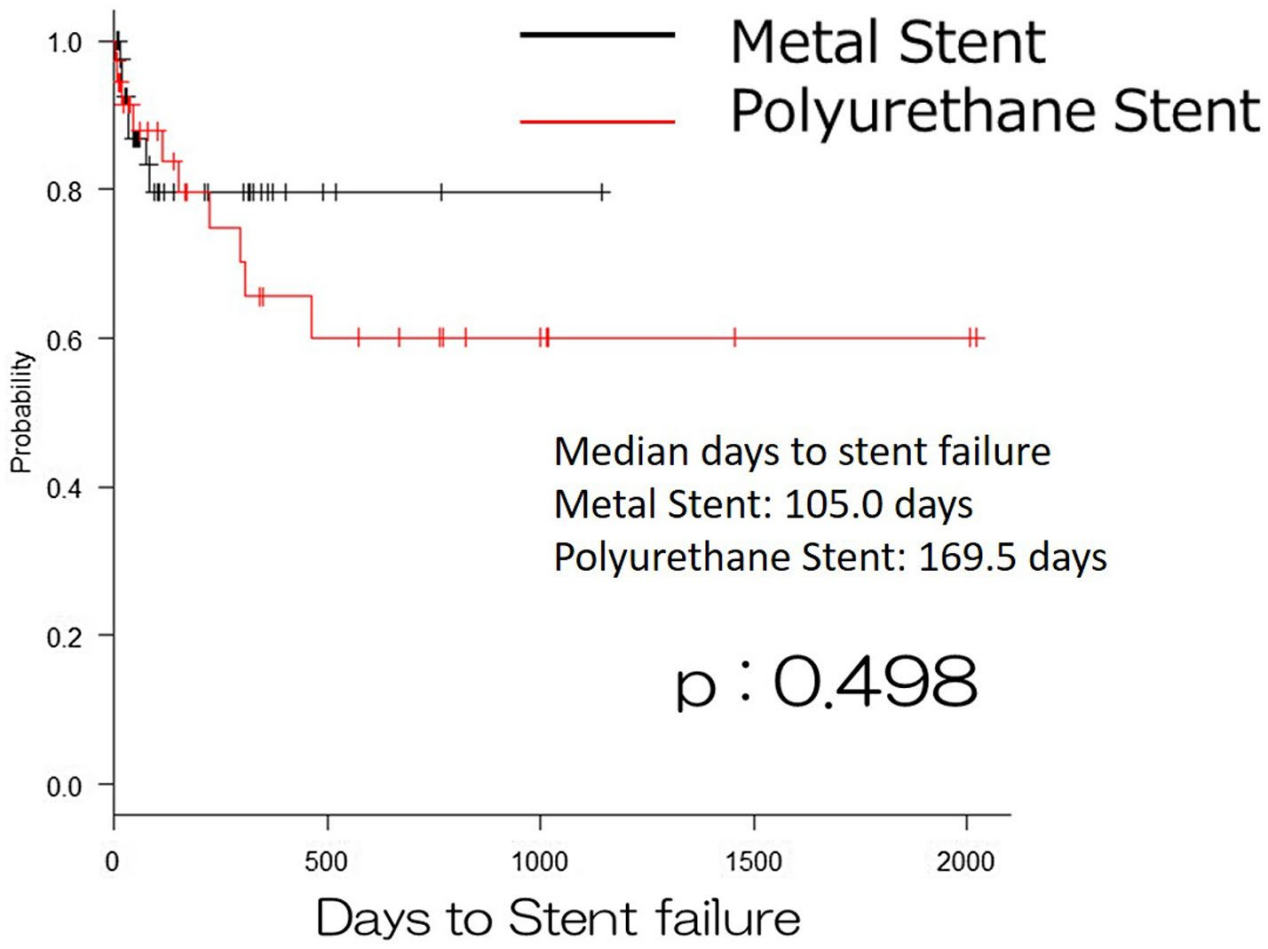
Stent failure free survival in patients with metal stent or polyurethane stent

A Kaplan–Meier analysis and log‐rank test indicated that there were no significant differences between the two groups in the overall survival (*P* = .673; Figure 1). It also indicated that there were no significant differences between the two groups in the rate of stent failure (*P* = .498; Figure 2).

### 3.2 Complications analyses

The frequency of complications is summarized in Table 2 (n; total number). One or more complications were experienced by 19 (32.2%) patients in the metallic ureteral stent group and 18 (38.3%) patients in the control group (*P* = .409). The types of complications are summarized in Table 3 (n; total number). Renal dysfunction after the replacement of a ureteral stent was experienced in 9 (15.3%) patients in the metallic ureteral stent group and 14 (29.8%) patients in the control group. There were no patients who had a UTI that required hospitalization in the metallic ureteral stent group, whereas 3 (6.4%) patients in the control group required hospitalization for a UTI (Table 4).

**TABLE 2:** Outcome in patients with ureteral stent

	Polyurethane stent	Metallic stent
Outcome		
Alive	17 (47.2%)	24 (53.3%)
Dead	19 (52.8%)	21 (46.7%)
Stent failure		
Yes	10 (27.8%)	8 (17.8%)
No	26 (72.2%)	37 (82.2%)
Unschedule admission		
Yes	15 (41.7%)	14 (31.1%)
No	21 (58.3%)	31 (68.9%)

**TABLE 3:** Unscheduled visit or admission

Unscheduled visit or admission	Polyurethane stent	Metallic stent	*P*‐value
	n (%)		
None	29 (61.7%)	40 (67.8%)	.409
One time	15 (31.9%)	17 (28.8%)	
Two times	1 (2.1%)	2 (3.4%)	
Three Times	2 (4.3%)	0 (0.0%)	
Total（≥1)	18 (38.3%)	19 (32.2%)	

**TABLE 4:** Complications of ureteral stent

	Polyurethane stent	Metal stent
Outpatient		
Irritation	3 (25.0%)	3 (27.2%)
Hematuria	1 (8.3%)	2 (18.2%)
Flank pain	1 (8.3%)	1 (9.1%)
Infection	0 (0.0%)	2 (18.2%)
Stent exchange		
Pain	1 (8.3%)	0 (0.0%)
Renal dysfunction	5 (41.8)	0 (0.0%)
Admission		
Making nephrostomy		
Infection	0 (0.0%)	1 (10.0%)
Renal dysfunction	9 (75.0%)	9 (90.0%)
Infection	3 (25.0%)	0 (0.0%)

A univariate analysis revealed that the age (<60 vs ≥60 years; *P* = .017) was associated with complications, whereas the gender, type of cancer, pre‐stenting, and history of chemotherapy and radiotherapy were not associated with complications. A multivariate analysis revealed that no factors were associated with complications (Table 5).

**TABLE 5:** Multivariate analysis for unscheduled admission

	Odd ratio	Lower	Upper	*P*‐value
		95% CI		
Age 60 yrs. or more	0.27	0.06	1.16	.078
Female	1.70	0.33	8.79	.529
Prestenting	2.87	0.28	29.30	.373
Chemotherapy	2.83	0.65	12.30	.167
Radiation therapy	2.48	0.51	12.00	.259
Local invasion	0.48	0.12	2.00	.312
Gastrointestinal cancer	10.69	22.00	6.00	.506

### 3.3 Cost analyses

The cost of placing metallic and polymeric ureteral stents for 2 years is 340 000 yen ($3000 US) and 403,200 yen ($3560 US), respectively, and the cost for 2.5 years is 510 000 yen ($4500 US) and 518 400 yen ($4580 US), respectively. While placement of a metallic ureteral stent is cheaper than that of a polymeric ureteral stent for 2 years, this difference is negligible at 2.5 years. The differences between 2.0 and 2.5 years were came from the period that metallic ureteral stenting was performed every 1 year. The total cost may vary depending on whether the patients made an unscheduled emergency visit or unscheduled ureteral stent exchange or making PCN.

### 3.4 Workload analyses

**FIGURE 3:**
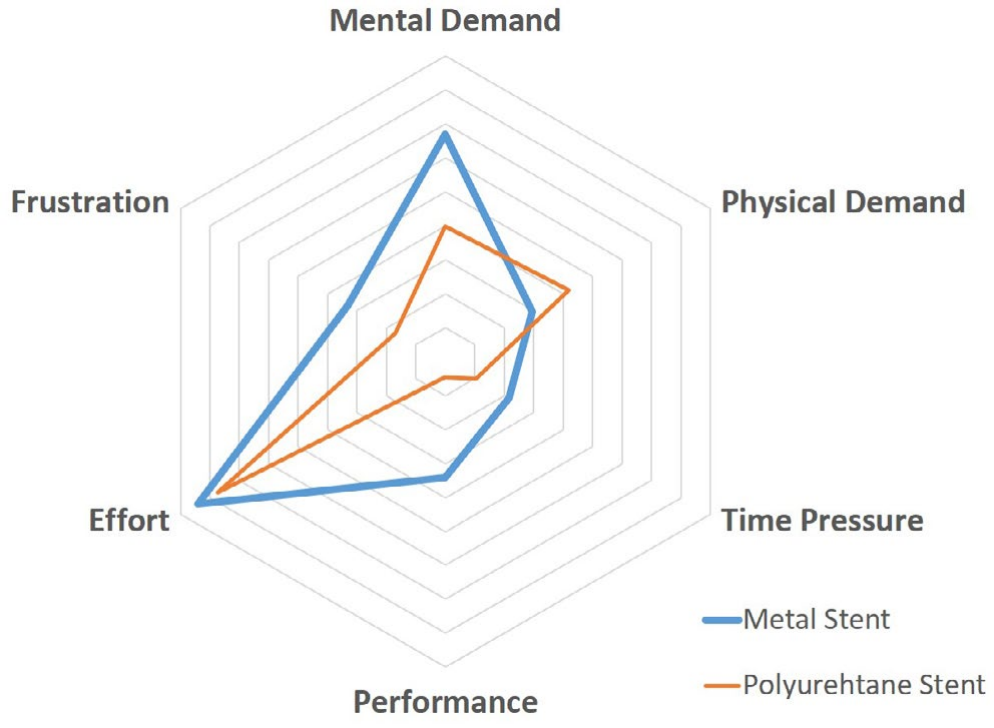
Need the effort for inserting ureteral stent

We sent out questionnaires to 11 urologists experienced in replacement metallic ureteral stents. All urologists answered this questionnaire experienced metallic ureteral stenting more than five cases. The average workload score in six subscales was analyzed (Figure 3). The mental demand and performance scores were higher for metallic ureteral stent, but there was no significant difference between metallic and polymeric ureteral stents.

## 4 DISCUSSION

In 2006, Borin et al. reported the first successful placement of a metallic ureteral stent in a patient with retroperitoneal fibrosis resulting from metastatic breast cancer. The metallic ureteral stent provided sufficient kidney drainage for 4 months.^21^

Several subsequent studies have reported that the mean follow‐up duration ranged from 5 to 12 months, with no major complications. The patency rate ranged from 46% to 100%.^2^, ^22^ While studies of metallic ureteral stents have involved fewer patients than those of polymeric ureteral stents, the patency rate was higher.^15^ The safety and efficacy of metallic ureteral stents has been recognized. Although some studies have reported outcomes of metallic ureteral stents, few have compared outcomes between metallic and polymeric stents. However, Po‐Ming et al. confirmed the superior efficacy of the metallic ureteral stent by comparing the sequential functional durations of different stents.^18^ They reported that stent‐related symptoms were similar between both kinds of stents, but metallic ureteral stents provided a longer functional duration than polymeric ureteral stents.

This study has several limitations as follows. The first one is that the number of urologist to answer NASA‐TLX is small. On the contrary, in our institute, the procedure were standardized using the lecture by expert and all urologists experienced metallic ureteral stenting more than five cases. Further study is needed to confirm which subject was affected between polyurethane ureteral stenting and metallic ureteral stenting. The second one is this study could not reveal the differences of complications between young and elderly. Younger age (<60) group showed significantly higher than elderly (≥60) group. In these patients, 5 of 11 (45.5%) were gynecological cancer. Thus, females with gynecological cancer showed irritation in higher percentage.

## 5 CONCLUSION

Metallic ureteral stents showed favorable ureteral stent patency and helped reduce the workload for urologists.

## CONFLICT OF INTEREST

We declare no conflicts of interest.

## ETHICS APPROVAL AND CONSENT TO PARTICIPATE

This study was approved by the ethics committee of Yokohama City University Medical Center. Approval number is B180900072.

## Data Availability

Due to ethical restrictions, the raw data that were used in this study are available upon request from the corresponding author.
